# A Review on Adventitious Lactic Acid Bacteria from Table Olives

**DOI:** 10.3390/foods9070948

**Published:** 2020-07-17

**Authors:** M. Francisca Portilha-Cunha, Angela C. Macedo, F. Xavier Malcata

**Affiliations:** 1 LEPABE—Laboratory for Process Engineering, Environment, Biotechnology and Energy, Faculty of Engineering, University of Porto, Rua Dr. Roberto Frias, 4200-465 Porto, Portugal; mfcunha@fe.up.pt (M.F.P.-C.); fmalcata@fe.up.pt (F.X.M.); 2 UNICES-ISMAI—Research Unit in Management Sciences and Sustainability, University Institute of Maia, Av. Carlos Oliveira Campos, 4475-690 Maia, Portugal

**Keywords:** olive cultivar, *Lactobacillus*, *Enterococcus*, *Pediococcus*, *Leuconostoc*, Mediterranean diet, processing method/trade, starter culture

## Abstract

Spontaneous fermentation constitutes the basis of the chief natural method of processing of table olives, where autochthonous strains of lactic acid bacteria (LAB) play a dominant role. A thorough literature search has unfolded 197 reports worldwide, published in the last two decades, that indicate an increasing interest in table olive-borne LAB, especially in Mediterranean countries. This review attempted to extract extra information from such a large body of work, namely, in terms of correlations between LAB strains isolated, manufacture processes, olive types, and geographical regions. Spain produces mostly green olives by Spanish-style treatment, whereas Italy and Greece produce mainly green and black olives, respectively, by both natural and Spanish-style. More than 40 species belonging to nine genera of LAB have been described; the genus most often cited is *Lactobacillus*, with *L. plantarum* and *L. pentosus* as most frequent species—irrespective of country, processing method, or olive type. Certain LAB species are typically associated with cultivar, e.g., *Lactobacillus parafarraginis* with Spanish Manzanilla, or *L. paraplantarum* with Greek Kalamata and Conservolea, Portuguese Galega, and Italian Tonda di Cagliari. Despite the potential of native LAB to serve as starter cultures, extensive research and development efforts are still needed before this becomes a commercial reality in table olive fermentation.

## 1. Introduction

Olives are drupes characterized by bitter taste, low carbohydrate content (2.5–6% *w*/*w*), and high oil content (10–15% *w*/*w*), depending on time of the year and cultivar [1]. Fiber justifies between 50% and 80% of those carbohydrates, whereas glucose, fructose, sucrose, xylose, and rhamnose account for the remainder. Despite a lower richness in sugars than other drupes, olives can still support active microbial fermentation, provided that sufficiently long timeframes are allowed. The aforementioned bitterness is due to the presence of phenolic compounds, chiefly oleuropein—which appears to play a defense role against predators. After a number of physicochemical and microbiological transformations, the said drupes will become edible and appealing in sensory terms [2]; this includes debittering as a result of conversion of oleuropein to hydroxytyrosol, both of which are strong antioxidants [3].

The main natural method of table olive processing is fermentation, which starts spontaneously owing to the presence of native microorganisms. The underlying microbial ecosystem is complex and variable; its dynamics throughout fermentation is determined by parameters that influence metabolism, namely, the indigenous microbiota itself, further to a number of intrinsic and extrinsic factors [4]. All in all, the quality of the final product remains variable and hard to anticipate.

In attempts to improve the process of plant product fermentation, starter cultures have been developed—and some are commercially available; however, these are not specific for table olives. More defined microbial populations for each cultivar of table olives are accordingly to be sought, namely, based on the best performant, native strains of lactic acid bacteria (LAB)—obtained directly from unique regional ecosystems, and selected for their functional (and probiotic) features. The resulting cultures would likely facilitate preparation of the corresponding Cahier d’Écharges and, eventually, support granting of a Protected Designation of Origin (PDO) status [5].

Fermentation of olives by autochthonous strains of LAB has traditionally been taken advantage of toward their preservation over long periods, using low pH brines; this indeed prevents the growth of unwanted microorganisms, as per ecological dominance. On the other hand, the market for table olives has been steadily growing; it has now expanded to non-producing countries [6,7] because of the increasing popularity of the Mediterranean diet as a healthy diet (and table olives are key ingredients thereof). Furthermore, table olives may be viewed as value-added products because of their content in nutraceutical and probiotic ingredients—as indicated by several scientific studies; hence, table olives are also emerging as a promising functional food.

Given the above, this review has attempted to compare and critically discuss LAB strains isolated from various preparation processes in several Mediterranean countries—with the aid of statistically based tools for a more objective analysis.

## 2. Processing Methods of Table Olives

**Figure 1:**
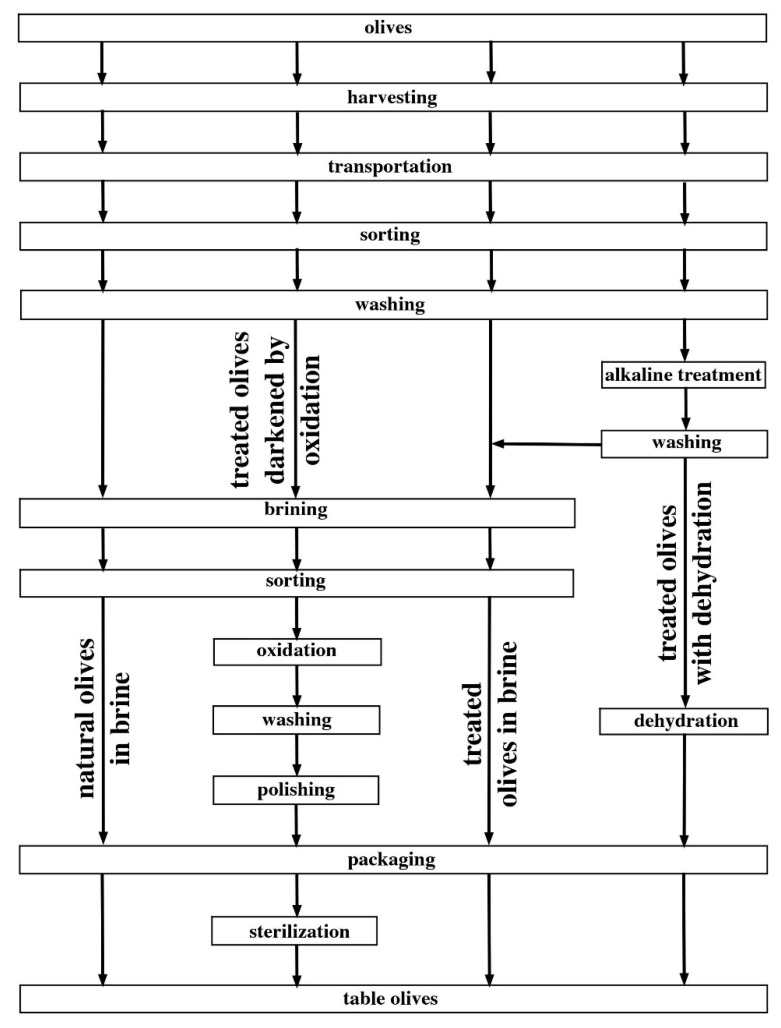
Main steps of widespread processing methods for table olives.

In the Trade Standard Applying to Table Olives [2], the International Olive Council (IOC) summarized the types of existing table olives—green olives, olives turning color, and black olives according to degree of ripeness of the fresh fruits; and their trade preparations—treated olives, natural olives, dehydrated and/or shriveled olives, olives darkened by oxidation, and specialties. The key aspects of the generic trade preparations of table olives are summarized in Table 1. To better understand the similarities/differences among the various processes available, these are laid down in parallel in Figure 1—from raw materials to final products.

As apparent from inspection of Figure 1, table olives must undergo a series of physicochemical and microbiological transformations before turning fully edible. Their natural bitterness can be removed via general-purpose, ill-defined methods—such as brine fermentation, heat treatment, treatment with dry salt, and plain immersion in water or by specific chemical methods such as alkaline treatment with sodium hydroxide [2]. Afterward, olives are preserved in brine according to their specific characteristics or in dry salt under modified atmosphere, upon addition of preservative or acidifying agent(s) or after pasteurization/sterilization.

Regarding natural olives, all three types are placed directly in brine, without alkaline treatment, and subsequently preserved as such or after addition of acidifying agents. These approaches are typical in Eastern Mediterranean countries, and their popularity derives from the slightly bitter taste and aroma attained, in the absence of any specific chemical attack [1]. Despite their increased importance in the market, natural olives require a longer processing period, more effort and energy, and undergoing a more considerable loss of mass than competing, fully industrialized processes—which hamper economic feasibility [8]. Bitterness, in particular, is reduced via fermentation—yet this process takes time, since diffusion of soluble components through the epidermis of the original fruits is slow when alkaline treatment has not been applied in advance. This trade preparation is also known as Greek-style when applied to black olives; although color may fade away, oxidation a posteriori returns them to their original dark color [9].

On the other hand, all three olive types can be processed via the trade preparations: olives may undergo alkaline treatment (with an ideal concentration of sodium hydroxide dependent on degree of ripeness, cultivar, temperature, and desired rate for penetration/leaching of organic molecules); be packaged in the brine where fermentation took place; and be added (or not) with acidifying agents in the end to extend their shelf-life [9]. Nowadays, one of the trade preparations implemented at a larger scale is Spanish-style (or Sevillian) green olives. Complex and variable microbiota typically arise during fermentation thereof, which change over time. Controlling the salt percent and assuring a low pH (<4.0) at packaging are important to eliminate unwanted microorganisms and avoid various forms of deterioration. Another example is Picholine-style green olives [1]—which do not resort to fermentation, since bitterness is removed by the alkaline treatment. Temperature control plays a relevant role though, because high temperatures likely destabilize the product [9].

Olives darkened by oxidation are also known as Californian-style [1]. Green or turning color olives can be directly processed or, more often, preserved in brine (where fermentation took place) before oxidation. This step is essential to correct color fading caused by the alkaline treatment—since a uniform and stable black color is a must; and it can be accomplished through injection of air or addition of iron salts. Moreover, these olives are to be packed in hermetically sealed containers and sterilized to guarantee product safety and avoid growth of pathogens and spoilage microorganisms [9].

Although all types of olives can be dehydrated, the most common are black olives, typically of Greek origin, added with dry salt. They may or may not be subjected to mild alkaline treatment; in either case, they are vigorously washed and placed in baskets with alternating layers of dry salt—to a final concentration of approximately 15%(w_salt_/weight_olives_) [9]. Alternatively, dehydration can be brought about by supplying heat. The final product is salty rather than bitter and is ready for local consumption [1].

Finally, specialties are olives that can be prepared using various processes or as a complement to the preparations tabulated in Table 1. They retain the designation “olive” as long as the fruits utilized comply with the general definitions set by the Trade Standard Applying to Table Olives [2].

Although olives typically undergo one of the above four trade preparations, a very many unique processing methods exist in the world that have proven successful [9]—normally a result of knowledge accumulated over sequential generations of empirical work. Most such alternative processes are of regional or local nature and directly reflect the importance of terroir; many of them are available only at the household scale.

With countless types of olives available on the market, consumer preference serves as a competitive advantage; nevertheless, scarce studies have focused on consumers’ behavior—in terms of needs and expectations when buying table olives. It is easy to understand how sensory properties, such as color, appearance, odor, taste, texture, saltiness, hardness, and crispness, can significantly influence consumers’ choices; however, cultural practices, packaging, and socioeconomic status (among others) also play a role—more subjective by nature and, thus, more difficult to anticipate [10,11,12]. In any case, consumers are more prone to purchase table olives processed according to regional methods; for instance, green Spanish-style olives are the most popular amongst Spanish consumers, while naturally black ripe olives in brine or salt-dried olives are predominant in Greek markets, and Californian-style black olives are mostly sought in the USA [13].

## 3. Fermentation of Table Olives

### 3.1. Spontaneous Fermentation

Fermentation may start spontaneously as brought about by autochthonous microorganisms; such a microbial ecosystem is, however, complex, variable, and often ill-defined. The dynamics of fermentation is determined by parameters that influence metabolism—namely, the indigenous olive microbiota itself which depends on cultivar, degree of ripeness, region, and farming practices; Enterobacteriaceae, *Pseudomonas*, *Leuconostoc*, *Pediococcus*, *Lactobacillus*, yeasts, and molds are the most common microorganisms found. A metagenomic analysis encompassing adventitious microbiota and olives unfolded *Lactobacillus*, *Celerinatantimonas*, *Propionibacterium,* and *Pseudomonas* as the most relevant genera; communities of yeasts and fungi are less diverse, and dominated by *Pichia*, *Ogataea*, and *Saccharomyces* [14]. This systematic analysis of Kalamata cultivar also supported biogeographic discrimination of samples (between Messinia/Lakonia and Aitoloakarnania). An “omics” approach to the whole fermentation process of natural green Aloreña de Málaga table olives—resorting to a high-throughput, barcoded pyrosequencing analysis of V2–V3 hypervariable region of bacterial 16S rRNA gene, showed 97% identity of 131 bacterial genera included in 357 operational taxonomic units [15]; the bacterial biodiversity was higher at the beginning of the process and decreased considerably as time elapsed. Enterobacteriaceae and Lactobacillaceae were scarcely found, unlike the high frequency of *Celerinatantimonas* spp. (95% in brine and 89% in olive samples); along with *Pseudomonas* and *Propionibacterium*, besides halophilic bacteria (viz. *Modestobacter*, *Rhodovibrio*, *Salinibacter*) identified as spoilage vectors—while pathogens were absent. Furthermore, intrinsic factors of the fruit, such as pH, water activity, nutrient availability, resistance to diffusion (dependent on olive skin and pulp structure), and levels of antimicrobial compounds released (such as oleuropein and other phenolic compounds), play a role; as well as extrinsic factors such as temperature, salt concentration, and oxygen availability in the brine [4,16,17]. Yeasts are considered next to LAB in degree of importance toward table olive fermentation. They may even enhance LAB growth, namely, for degrading phenolic compounds that are inhibitory to such bacteria; while producing volatile metabolites that enhance sensory features. Conversely, they can contribute to soften the olive tissue and form gas pockets due to the CO_2_ production at an early stage—both of which compromise final product acceptability [18,19].

Bitterness can be removed, namely, via lye treatment as happens in the green Spanish-style; this process also removes other inhibitory phenols, prior to environmental contamination or deliberate inoculation by LAB. When a chemical method is not used, debittering relies solely on LAB-mediated fermentation which also contributes to the synthesis of other compounds with a role in flavor development. This justifies why LAB have become a quite (probably the most) important group of bacteria in table olive processing. In addition, these microorganisms can acidify the brine (essential to maintain a low pH) and produce antimicrobial compounds (e.g., bacteriocins)—both relevant to prevent growth of unwanted microorganisms (e.g., *Escherichia coli* O157:H7, *Listeria monocytogenes*, or *Clostridium botulinum*); besides taking advantage of an ecological dominance mechanism, stemming from their initial numbers, and rate of multiplication [18,19]. Assessment of malodorous spoilage of Spanish-style green Gordal and Manzanilla table olives has been conducted by Castro [20], under low salt concentration (approximately 7% NaCl); based on high-throughput DNA sequencing, *Cardiobacteriaceae* and *Ruminococcus* were found as dominant bacteria. Degradation of lactic acid and a significant increase in volatile fatty acid and phenol contents were found, as compared to (unspoiled) control samples; good correlations were reported between microbial communities, metabolites, and sensory descriptors—e.g., the “zapatera” descriptor was significantly associated with *Propionibacterium* and positively correlated with acetic, propionic, succinic, and methyl propanoic acids, while the “butyric” descriptor was significantly associated with *Ruminococcus* and positively correlated with propionic and butyric acids.

### 3.2. Controlled Fermentation

Selected starter cultures are widely used in a few food sectors, such as alcoholic beverages and dairy products but not routinely used in table olive fermentation [19]. In fact, this type of process is still mainly craft-based and empirically driven by the native microbiota present on olives, thus leading to a final product of variable (and often unpredictable) quality. This topic has been extensively tackled in the past few years, with emphases on the characteristics and advantages of using selected starter cultures [19,21]; desirable and undesirable characteristics of microorganisms meant for use as starter cultures [19]; and main bacterial and yeast species present (alone or in combination) [21]. Among the most important factors that constrain correct development of LAB-mediated fermentation is fruit pH, and phenolic compound and nutrient contents, efficiency of previous rinsing (namely, in terms of residual alkali left), level of salt in the brine, processing temperature, and vat aeration degree [22]. Organic acids can be added to the brine to establish an optimal pH for LAB growth, whereas salt concentration has to be carefully controlled at the beginning of the process and upon every brine renewal; lower NaCl levels typically promote LAB development, whereas higher values favor yeasts [23]. Despite its relevance, the temperature is difficult to control—and depends on vat size; cultivar and maturity stage at harvest normally constrain phenolic compound and sugar contents as well as cell wall permeability. Some LAB appear to develop better in the presence of yeasts [24] which, when utilized as starter adjuncts, inhibit both undesirable contaminating yeasts and foodborne pathogenic bacteria [25].

Industrial microbiology has been making use of microorganisms, such as naturally occurring ones, laboratory-selected mutants, or genetically modified organisms, to produce a wide variety of industrial products of human interest [26]. Natural strains, following previous isolation and multiplication, have already been used as standard starter cultures for table olives; several pros and cons are presented elsewhere [5]. The chief advantages relate to their wide biodiversity—as they are made up of microorganisms that spontaneously colonize the raw materials and contribute as a whole to enrich the final product with unique sensory keynotes [27]; their addition also facilitates control over aroma, texture, and flavor of the final product [28]. All in all, most authors have concluded that the use of starter cultures justifies better processes/products than carrying the fermentation at the expense only of adventitious microflora [22]—in safety and sensory terms. In recent years, an increase in the frequency of application of autochthonous strains to the manufacture of table olives has been witnessed, despite the scarce scientific studies available thereon [5]. This trend has surfaced in attempts to deal with consumers’ demand for more traditional and homemade products, exhibiting unique sensory profiles and health benefits, and resorting to non-dairy foods as vehicles [15,29].

Starter cultures not only decrease the risk of spoilage but also aid in acidifying the brine [22]; an ideal starter culture should grow fast even at relatively low temperature, entail homofermentative metabolism, high acidification rate, and fast nutrient depletion, and possess good tolerance to salt, organic acids, and phenolic compounds. To avoid disrupting the tissue structure of olives that would eventually compromise their texture at consumption time, previous thermal treatment to get rid of adventitious microflora is not recommended; effective preliminary rinsing usually suffices—provided that sufficiently large LAB inocula are provided. An “omics” approach was followed by de Angellis [30] to ascertain the ability of selected lactobacilli and yeasts to improve the fermentation process of Bella di Cerignola table olives. Four distinct batches were tested: (i) commercial *Lactobacillus plantarum* (S), (ii) commercial *L. plantarum* and autochthonous yeast *Wickeramomyces anomalus* DiSSPA73 (SY), (iii) commercial *L. plantarum*, autochthonous *W. anomalus* DiSSPA73, autochthonous *L. plantarum* DiSSPA1A7, and autochthonous *Lactobacillus pentosus* DiSSPA7 (SYL), and (iv) a starter-free batch (Ctrl). Unfermented olives and olives by one day of fermentation were composed solely of (Enterobacteriaceae) *Hafnia alvei* and *Methylobacterium* sp; conversely, *L. plantarum* and *L. pentosus* dominated the metabolically active microbiota of Ctrl brines and olives by the end of fermentation. The number of species and the results of an alpha-diversity analysis unfolded a marked simplification of microbial diversity in S, SY, and (chiefly) SYL batches; the lowest abundance was accounted for by Proteobacteria, including Enterobacteriaceae, *Lactococcus lactis*, *Propionibacterium acidipropionici*, and *Clostridium* spp. The lactobacilli and *W. anomalus* DiSSPA73 used in this study substantially affected the amounts of free amino acids, phenolics, and volatile organic compounds. A textural profile analysis and a sensory panel both showed the highest appreciation for table olives manufactured with starter [30]. A metagenomic approach to the microbial profiles of Picual olives indicated meaningful differences between spontaneous and inoculated treatments [31]; use of a starter shortened fermentation time considerably and olive debittering ending up in hydroxytyrosol was also accelerated; Enterobacteriaceae vanished faster as well. The process could be standardized, and an improvement was noted in the final products regarding functional and sensory properties.

The effect of distinct salt concentrations upon physicochemical, microbiological and sensory features, and volatile organic compound profile was evaluated in Nocellara Etnea table olives [32]. Latic acid bacteria dominated the microbiota from the 7th day of fermentation on; reduction of yeast and enterobacterium numbers were also observed in the final product, when either starter was employed. A large number of aldehydes at the beginning of the process is to be outlined, which decreased significantly afterward—with a concomitant increase of alcohols, acids, esters, and phenols. Esters, in particular, were significantly more concentrated in experimental samples than in the control, thus justifying the more pleasant flavors found. Moreover, acetic acid, ethanol, and phenols—usually associated to off-flavors, correlated negatively with mesophilic bacteria and LAB. Note that salt content did not affect starter culture performance at all and slightly influenced the metabolome of table olives [32].

Nowadays, the market of functional foods is seeking new non-dairy probiotic foods, such as fruits and vegetables, due to the increase in incidence of lactose intolerance, concerns over excessive cholesterol, and spread of vegetarian and vegan lifestyles [33,34]. A few studies have meanwhile shown that table olives are quite promising in this deed. This is due to the fact of their intrinsic richness in vitamin E and oleuropein, both strong antioxidant agents; the latter can retain its nutraceutical role, even upon conversion to hydroxytyrosol effected by fermentation [35]. Table olives also serve, together with the brine, as carriers of adventitious (or deliberately) added strains with probiotic features [19,29,36]—owing to the microporous structure of their pulp and the suitable composition of the medium. Olives have long been known also for their richness in unsaturated fatty acid residues—approximately 75% of their oil inventory consists of ω-9 oleic acid residues; this compound disturbs intracellular signaling pathways involved in cancer cell development, prevents age-related metabolic syndrome and neurodegenerative disorders, and enhances the insulin signaling pathway required by glucose uptake from the blood [3]. Germane oligoelements present are Fe—necessary for the synthesis of red blood cells; copper—which decreases the risk of heart attacks; and calcium—essential for bone, muscle, and nerve health. Data encompassing specifically probiotic effects of strains isolated from table olives have been reviewed in depth elsewhere [21].

## 4. Importance of LAB from Table Olives

### 4.1. Selection of Relevant Publications

A thorough literature search was performed on SCOPUS, ScienceDirect, and PubMed with combination of “table olives” with “lactic acid bacteria”, “LAB” or “*Lactobacillus*” as search terms. Only articles or reviews written in English and published after 2000 were retained. Duplicates were removed and contents were checked to validate their relevance. A few publications quoted in their reference list were later sought and added to the database whenever germane. Publications on table olives, focused on yeasts but also including data on LAB, were included as well.

### 4.2. Current Relevance

From the aforementioned search, a total of 197 publications were kept. Only 11 were categorized as reviews, so most effort was applied to experimental production of new knowledge in this topic. A broad range of objectives were addressed, namely: characterization of table olive microbiota [5,19,21,22,28,37,38]; improvement of table olive processing methods, preservation, and shelf-life [5,21,38]; selection, development, and implementation of (multifunctional) starter cultures [5,16,19,21,22,28,38]; and selection and use of potentially probiotic strains [5,19,21,28,37,38]. Other topics tackled included physicochemical and nutritional characteristics of table olives, microbial alterations during processing, and isolation, characterization, identification, and genomic sequencing of microbiota [29,37,39,40].

**Figure 2:**
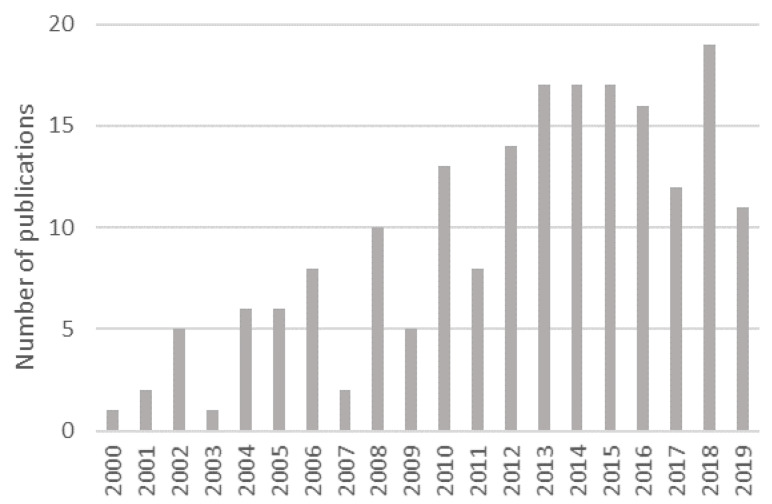
Publications per year on table olives that address lactic acid bacteria (LAB).

Research on this topic remains quite active, and a significant increase in the production of scientific documents over the years can be grasped in Figure 2; the vast majority have indeed been published after 2010. This trend correlates with market growth: table olive production increased from 2.08 million tons in 2008/2009 to 3.28 million tons in 2017/2018 [6], thus accounting for a 58% raise. Such an expansion seems primarily associated with the increasing popularity of the Mediterranean diet among the general public, and more frequent prescription thereof by health professionals; in fact, it is believed to bring about numerous health benefits which, as a whole, promote increased longevity and lower incidence of chronic diseases [41]. Table olives are themselves rich in monounsaturated fats and phenolic compounds—which function as antioxidants in the human body [16]. Furthermore, table olives are a proven source of relevant nutrients and offer a wide variety of aromas and flavors that support many culinary possibilities—most of which are much appreciated and valued by consumers [42]. An increased market demand for value-added products, such as nutraceutical or probiotic ingredients, has also supported table olives as an appealing functional food.

**Figure 3:**
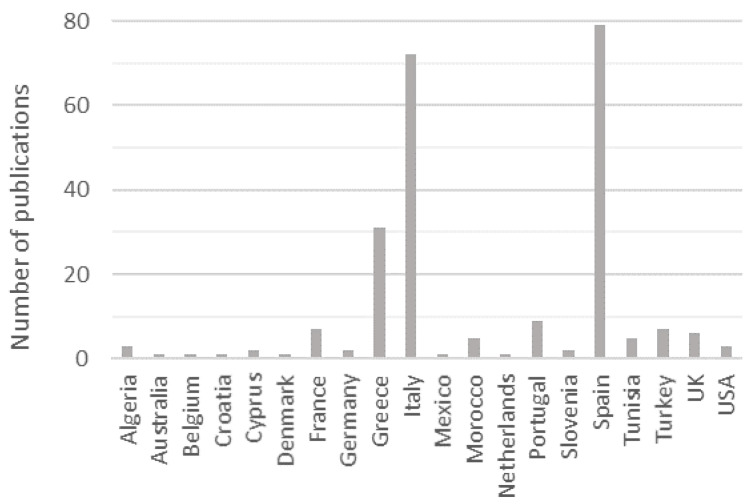
Publications per country on table olives that address lactic acid bacteria.

Our search confirms that most information on this topic has originated in countries located in the Mediterranean basin which also account for the most market share of table olives. Of the 3.28 million tons of table olives produced in 2017/2018, the largest individual producing countries were: Egypt (22.8%), Turkey (13.7%), Algeria (9.2%), Morocco (4.0%), and Syria (3.0%); and, within the European Union, Spain (17.1%), Greece (7.9%), Italy (1.8%), and Portugal (0.8%) [6,7]. These last four countries produced the most scientific publications as shown in Figure 3. Despite the market for table olives having expanded to non-producing or low-producing countries (where their popularity has meanwhile been increasing), not many studies have, to date, originated in such countries.

## 5. LAB Species Isolated from Table Olives

### 5.1. Compilation of Identified LAB Species

LAB are a group of bacteria able to perform lactic acid fermentation and can be divided in homofermentative (mainly producers of lactic acid) and heterofermentative (producers of lactic acid combined with ethanol and/or CO_2_) [43]. The most common LAB belong to genera *Lactobacillus*, *Leuconostoc*, *Pediococcus*, *Enterococcus*, and *Streptococcus*; however, *Aerococcus*, *Carnobacterium*, *Lactococcus*, *Oenococcus*, *Tetragenococcus*, *Vagococcus,* and *Weissella* have also been found [44,45].

Of the original articles identified in Section 4, the ones that tackled the identification of LAB species isolated from table olives were duly selected. From these, information pertaining to LAB species, olive cultivar, producing country, olive color, and processing method as well as the main objective of the study were retrieved. Due to the predominance of the genus *Lactobacillus*, there were more species identified of *Lactobacillus* than of all other genera combined together, the data collected are displayed in Table 2 and Table 3, separately for *Lactobacillus* and the remaining LAB, respectively.

As easily perceived from inspection of the first columns of Table 2 and Table 3, at least one species of the most common LAB genus has been isolated from all table olives; and more than 40 species belonging to nine genera have been reported. Second to *Lactobacillus*, the most frequent genera of LAB are *Enterococcus*, *Pediococcus,* and *Leuconostoc*. The most cited species are *L. plantarum* and *L. pentosus*—and *L. brevis*, *L. coryniformis*, *L. paraplantarum,* and *Lc. mesenteroides* to a lesser extent. Upon inspection of these tables, one also finds that each species is frequently associated with more than one country, processing method, and cultivar. Therefore, further knowledge will, in principle, be generated upon application of an appropriate method of statistical analysis—as done later, in Section 5.2. Note that the tables in this section are meant to play the role of an educated database for the interested reader; this is why a column summarizing the major objectives of each study was included. Most studies were accordingly aimed at identifying specific LAB (e.g., [49,53,57,68,86]) and/or assessing microbial diversity (e.g., [46,60,62,70,78,96]). In many cases, they were also aimed at characterizing the isolated strains, in terms of physicochemical (e.g., [57,94]), technological (e.g., [84,98]), or probiotic (e.g., [65,71,91]) features. Other important goals of those studies were selection and application of starter cultures (e.g., [32,54,74,80,93]); and sensory analysis (e.g., [60,66]) and safety evaluation (e.g., [56,80]), to a much lesser extent.

Although *Lactobacillus* spp. are the most abundant in many table olive processing methods, their predominance may also result from more extensive investigation focused thereon—which necessarily leaves many other species unidentified. In fact, up to 2012, only one study had characterized isolates from table olives using molecular tools [61]. However, new species have been reported regularly in recent years; wider availability of more accurate identification methodologies (chiefly molecular genotyping), as well as existence of generically accessible and increasingly populated genomic sequence databases may account for this realization. Molecular methods and culture-dependent and -independent approaches have been reviewed previously [40]; even though the authors argued that application of next-generation sequencing and meta-analysis methods—such as metagenomics and metatranscriptomic, would allow in-depth analysis of isolated microorganisms, this research area still has a long way to go in what concerns table olive microbiota.

Findings reported in a group of studies pertaining to halophilic and alkaliphilic LAB (HALAB) [48,67,97] were deliberately not included in the above tables; this designation is indeed quite recent and not yet widely accepted, so the specific species identified have, to date, been poorly characterized. According to such studies, HALAB are part of the characteristic microbiota at the initial stage of Spanish-style table olive fermentation and could contribute to produce fermenting brines ready for growth of common LAB—since they can perform lactic acid fermentation under highly alkaline conditions, for being alkali-tolerant. Germane species found include *Alkalibacterium pelagium*, *Alkalibacterium psychrotoleran*, *Halolactibacillus* sp., and *Marinilactibacillus psychrotolerans/piezotolerans*.

As for the remaining tables, the reported species have been isolated from 26 cultivars (cvs.) from a total of nine countries. As established in Section 4.2., most information available pertains to Italy (12 cvs.) and Spain (4 cvs.)—while less than half of the cited cultivars belong to Algeria (3 cvs.), Greece (3 cvs.), Croatia (1 cv.), Portugal (1 cv.), Morocco (1 cv.), Turkey (1 cv.), and Cyprus (even though the cultivar studied was not specifically identified in this case). As for processing method, almost all entries refer to either natural or treated table olives—with approximately twice as many entries for natural trade preparation, irrespective of olive color. In fact, none of the reported species was isolated from dehydrated olives, and only one was isolated from olives darkened by oxidation/Californian-style (related to the Oblica cultivar from Croatia). The majority of treated olives were processed as per the Spanish-style method, whereas only one study applied the Picholine-style method (related to the Picholine cultivar from Morocco), and two others indicated that table olives had been processed merely by some treatment.

### 5.2. Statistical Analysis of LAB Species Data

Concerning the variables *Lactobacillus* species, olive color, processing method, and country of origin (see Table 2), our statistical analysis (see Table 4) has indicated that all variables are significantly correlated with each other (at a significance level of *p* < 0.01).

In view of the results depicted in Table 4, exploratory factor analysis (EFA) with Cramer’s V correlations, principal components extraction, and oblique rotation (direct oblimin) were carried out using IBM SPSS^®^ V.26 (IBM, Chicago, IL, USA) with Syntax for software.

The Kaiser–Meyer–Olkin (KMO) measure was utilized to check that sample size was adequate for our intended analysis; and Bartlett’s test of sphericity was used to ascertain the inter-correlations among variables. The KMO measure (0.678) indicated that sampling size behavior was indeed adequate for EFA, with variables exhibiting significant correlation to each other, according to Bartlett’s test of sphericity (*p* = 0.000).

**Figure 4:**
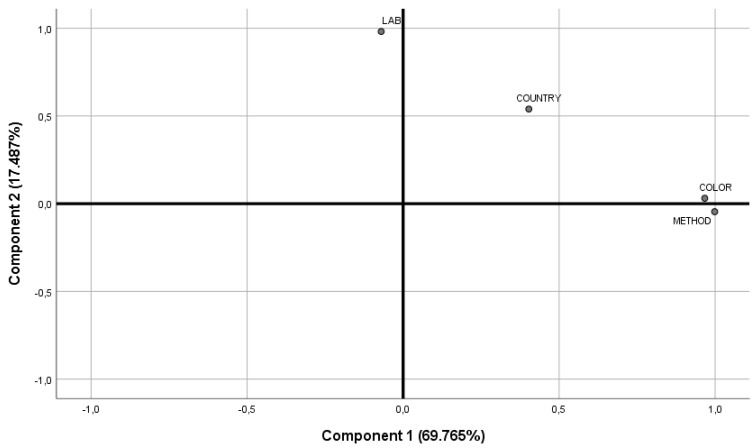
Plot of communalities from component extraction method, considering the variables country, color, and method type.

Two components were selected considering the scree plot—thus justifying 87.3% of total variance (see Figure 4). Component 1 was highly correlated to olive color (C1: 0.967, C2: 0.031) and method type (C1: 0.998, C2: −0.046), while component 2 was highly correlated to LAB species (C1: −0.070; C2: 0.981); country (C1: 0.403; C2: 0.540) appeared to correlate to both components.

**Figure 5:**
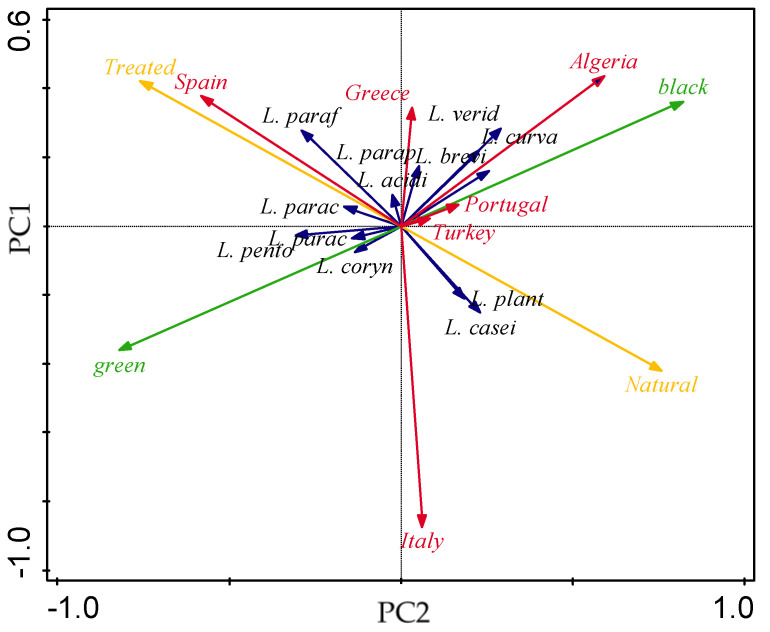
Plot of loadings formed by principle component analysis. LAB, country, color, and method type are represented in black, red, green, and yellow, respectively.

The plot of loadings and the plot of scores were obtained by Canoco™ V5.0 (Microcomputer Power, Ithaca, NY, USA). Inspection of the former (see Figure 5) indicated that green olives lie opposite to black olives and natural olives opposite to treated olives—as expected, for entailing somehow antagonist cultivars/processing modes. Algeria produces black olives by natural process; on the other hand, Spain produces green olives by Spanish-style (treated).

**Figure 6:**
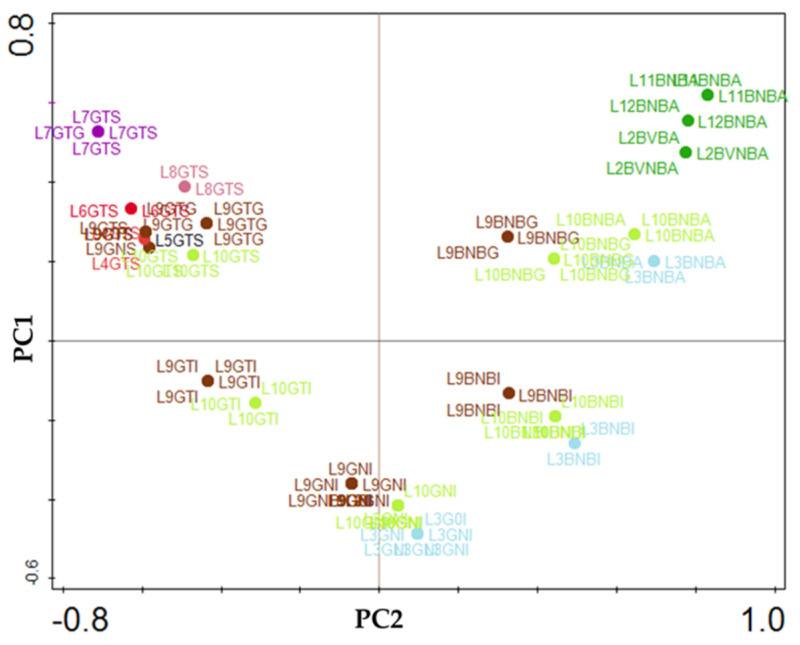
Plot of scores formed by principle component analysis, pertaining to LAB species (L1 = *L. acidipiscis*, L2 = *L. brevis*, L3 = *L. casei*, L4 = *L. coryniformis*, L5 = *L. paracasei* L6 = *L. paracollinoides*, L7 = *L. parafarraginis*, L8 = *L. paraplantarum*, L9 = *L. pentosus*, L10 = *L. plantarum*, L11 = *L. veridesens*, L12 = *L. curvatus*, L13 = *L. rapi* L14 = *L. rhamnosus*, L15 = *L. vaccinostercus*, L16 = *L. fermentum*), olive color (G = green, B = black), type of processing treatment (N = natural, T = treated), and country of origin (A = Algeria, G = Greece; I = Italy; P = Portugal, S = Spain, T = Turkey).

A plot of scores is conveyed in Figure 6, where seven clusters can be pinpointed. On the top-left side, *L. parafarraginis* was reported only for Spanish-treated green olives from the Manzanilla cultivar (see Table 2). On the top-right side, *L. brevis*, *L. curvatus*, and *L. veridesens* were typically associated with Algerian natural black olives, despite belonging to different cultivars (Chemlal, Hamra, and Sigoise). Additionally, *L. plantarum* and *L. pentosus* were found in the remaining five clusters; this means that such species are present in all types of olive cultivars reported, irrespective of country, processing method, or olive color. Finally, two clusters are apparent on the lower-left side; *L. casei* was typically found in natural processes with Italian green or black olives and Algerian black olives.

Concerning variables *Lactobacillus* species, processing method, and olive cultivar (see Table 2), our analysis of correlations (see Table 5) indicated that all variables were significantly correlated with each other (at a significance level of *p* < 0.05).

The KMO measure (0.611) indicated that sampling size behavior was indeed adequate for EFA with variables exhibiting significant correlation to each other, according to Bartlett’s test of sphericity (*p* = 0.000).

**Figure 7:**
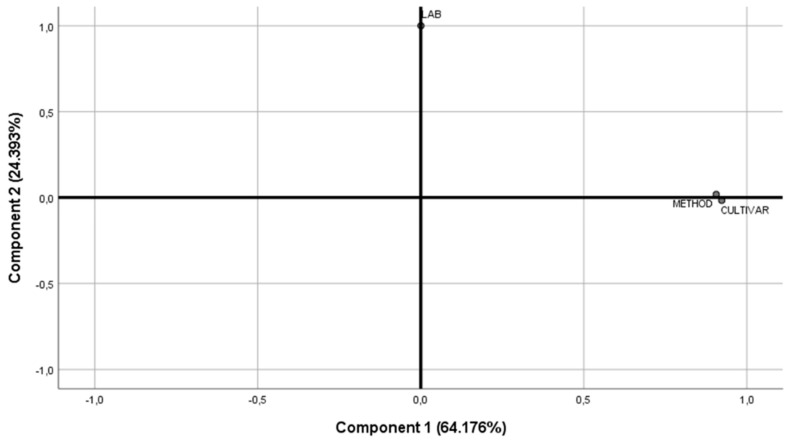
Plot of communalities from component extraction method, considering the variables LAB, cultivar, and method type.

Two components were selected considering the scree plot—thus justifying 89.1% of the total variance (see Figure 7). Component 1 was highly correlated to cultivar (C1: 0.906; C2: 0.018) and method type (C1: 0.923; C2: −0.017); while component 2 was highly correlated to LAB (C1: 0.000, C2: 1.000).

**Figure 8:**
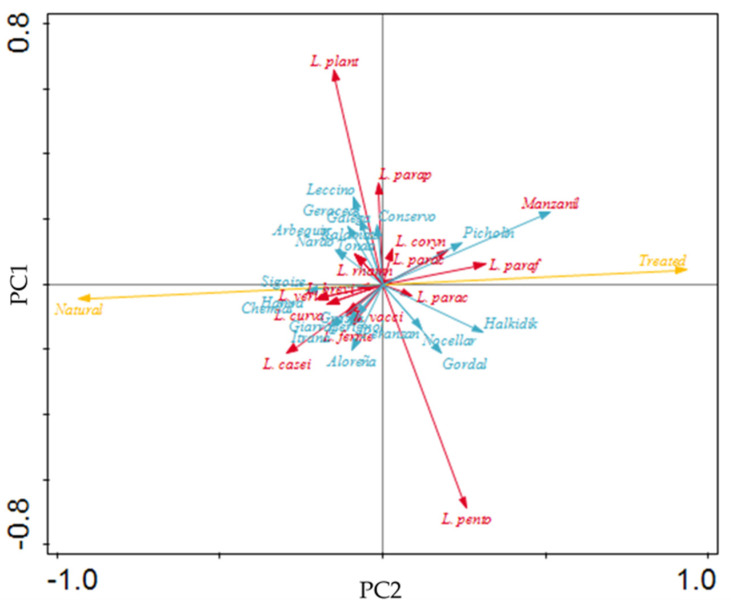
Plot of loadings formed by principle component analysis. LAB, method type, and cultivar are represented in red, yellow, and blue, respectively.

Inspection of the plot of loadings (see Figure 8) unfolds natural olives being opposite to treated ones—a trend referred to before; this serves as a double-check of the previous analysis. 

From inspection of Figure 8, correlations are clear between LAB species and cultivar: (i) *L. casei* and Itrana/Grossa; *L. fermentum* and Peranzana; *L. brevis*/*L.curvatus*/*L. veridenses* and Chemlal/Hamra/Sigoise; *L. paraplantarum* and Kalamata/Conservolea/Galega/Tonda di Cagliari; *L. paracasei* and Manzanilla/Halkidiki; *L. vacisnosterans* and Bella di Cerignola; and *L. parrafarragins*/*L. coryniformis* and Manzanilla.

**Figure 9:**
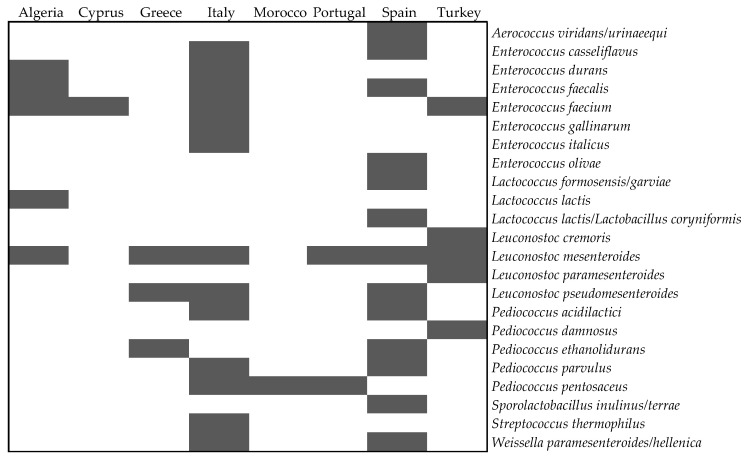
Geographic origin of identified LAB (*Lactobacillus* spp. excepted) from table olives. Grey indicates presence of species, and white indicates absence thereof.

The same methodology could not be applied to the remaining LAB species included in Table 3, for lack of case numbers. Figure 9 was instead prepared as a matrix of occurrences, intended to facilitate extraction of information in tabular form with entries by country and species.

Besides Italy, Spain, and Greece, almost no other LAB species were identified in the remaining table olive-producing countries. This fact may once more be explained by the fewer studies published on the current topic—and is not necessarily a consequence of smaller biodiversity of LAB associated with the olive cultivars assessed. Such an information gap leaves room for relevant scientific studies, particularly if/when producers want to improve their table olives processing system.

Within the reported LAB species in Figure 9, *Lc. mesenteroides* is the one associated with the highest number of countries, followed by *E. faecium* and *Lc. pseudomesenteroides*; however, most such species were isolated in only one or two countries. This trend of finding different pictures of biodiversity according to country seems to be linked to the specific cultivar studied and the processing method employed—among the numerous local and distinct methods available, which diverge significantly from country to country.

### 5.3. From Research Findings to Industrial Applications and Commercialization of LAB

Microbial starters, selected for specific biotechnological and safety traits, have been claimed [101] to perform a number of useful roles in table olive manufacture: (i) improvement of sensory attributes; (ii) better control of fermentation process; (iii) easier monitoring of process evolution; (iv) preservation and/or improvement of nutritional and healthy features of final product; (v) protection from undesirable spoilage and pathogenic microorganisms; (vi) fortification of the final product with microorganisms exhibiting probiotic potential; and (vii) enhancement of product stability and extension of shelf-life.

To be used in food fermentation, microorganisms should be selected at the strain level—since not all strains are equally suitable for use as starters, nor are all equally well-adapted to food substrates [102,103]. In fact, the selection of inappropriate strains may lead to negative results, such as production of undesirable metabolites or even misprocessing [19,104]. Selected strains must satisfy several characteristics [16,19,26] which can be grouped as intrinsic characteristics of the microorganism; technological characteristics; and safety and benefits for humans. Regarding the microorganism, the strain should: be genetically stable; easily adapted to the raw material and the fermentation environment; grown under the environmental conditions prevailing in the food and dominate the indigenous microbiota; possess specific enzymes that contribute positively to the final product; and be capable of protecting itself from contamination while not being vulnerable to bacteriophages. The main technological characteristics include growing in large-scale cultures and relatively inexpensive liquid culture media; producing the substance of interest in a relatively short period, thus allowing easy recovery; and resistance to freezing or freeze-drying processes. The benefits and safety characteristics can be summarized as not being harmful to humans, animals or plants, and having probiotic/health-promoting/disease-preventing effects. After being cultured, microorganisms are to be harvested, packaged, and stored under optimal conditions for growth, as well as survival.

**Figure 10:**
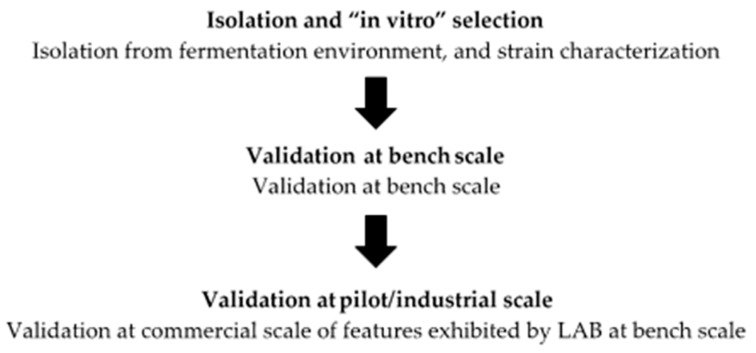
Major steps underlying the selection of starter cultures.

Selection and implementation of a starter culture is, nevertheless, a difficult matter in attempts to guarantee a successful fermentation. Despite the absence of specific rules to guide selection, the three main steps highlighted in Figure 10 are to be followed in general [19,105].

Although this review has focused on the first step, selected studies and reports concerned with validation, at laboratory/pilot/industrial scales, of LAB for table olive fermentation will be briefly discussed below—for the sake of completeness. Remember that *L. plantarum* and *L. pentosus* were the LAB species most often reported in the literature (see the Section 5.2); this realization holds irrespective of cultivar, olive color, country of origin, or processing method. Therefore, it comes as no surprise that most trials have taken place with such two species, at all size scales of the fermentation batch.

Laboratory-scale fermentation of table olives from Taggiasca cultivar was studied by testing the influence of two operational parameters: *L. plantarum* as the single starter, or in combination with *Saccharomyces cerevisiae*, at three temperatures (23 °C, 30 °C or 37 °C). The results confirmed the efficiency of starter-mediated treatments when compared with control tests, in terms of debittering of black table olives [106]. 

Pilot-scale trials showed that a rapid (yet controlled) fermentation process can be achieved by using brine with modified pH, and carbohydrate and growth factor concentrations, when in the presence of *L. pentosus* 1MO [107]. Three major findings evolved from this study: (i) addition of *L. pentosus* entails a useful tool to decrease the possibility of spoilage; (ii) growth rate of LAB and levels of the most important fermentation parameters (viz. lactic acid production and pH) are significantly improved when the brines are supplemented with glucose and yeast extract; (iii) *L. pentosus* 1MO plays a relevant role upon hydrolysis of oleuropein, and concentration of the dialdehydic form of decarboxymethyl oleanolic acid linked to hydroxytyrosol—while verbascoside remains unchanged, with a consequent reduction in the original bitter taste. This process was accordingly upgraded to industrial level; a related strain, *L. pentosus* OM13, belonging to the same species, was applied at large scale to Nocellara del Belice olives. This study provided strong evidence that addition of lactic acid, nutrient adjuvants, and (most importantly) acclimatization of LAB cells significantly shortens the acidification process in the olive brine and concomitantly improves safety and sensory quality of the final table olives [108].

A thorough search on the PATENTSCOP database (WIPO, Genebra), using “table olives” as keywords, unfolded sixty patents—of which only 14 pertained to table olive processing; however, only eight of those held distinct titles and originated from distinct authors. From those eight patents, four mentioned the addition of LAB to improve the process, but only two identified the LAB employed to the species level. More specifically, the invention by Bleve et al. [109] encompassed starter cultures comprising *S. cerevisiae* (cod. DSMZ 27800) and *L. plantarum* (cod. DSMZ 27925) for preparation of Italian Leccino cultivar olives; as well as *S. cerevisiae* (cod. DSMZ 27801) and *Lc. mesenteroides* (cod. DSMZ 27926) for preparation of Greek Kalamata cultivar olives; the deposit number codes refer to Leibniz-Institut DSMZ (Germany). The second outstanding patent referred to evaluation of colonization of the pericarp of table olives, and survival of the following probiotic strains: *L. rhamnosus* GG ATCC53103, *L. rhamnosus* IMPC 11 and IMPC 19, *L. paracasei* IMPC 2.1 and IMPC 4.1, *Bifidobacterium bifidum* ATCC 15696 and *Bifidobacterium longum* ATCC 15708—all deposited with the Belgian Coordinated Collections of Microorganisms [110].

A starter culture consisting of two *L. pentosus* strains was developed and tested for production in Spanish-style green olives—and has afterward been extensively utilized by the industry in Spain and other Mediterranean countries and even Argentina [111]. The medium-size enterprise Simantra (Chalkidiki, Greece), in turn, collaborated with an industrial scale study about the evolution of safety and quality parameters of Greek PDO green table olives Prasines Elies Chalkidikis—prepared according to the Spanish-style, over 12 months of storage, with two consecutive periods for the first stage [112]. The authors claimed that the final product was microbiologically safe and that most trade quality parameters were acceptable; attention should, however, be paid to the reduction in content of phenolic compounds (i.e., hydroxytyrosol, tyrosol) and to the significant increase in sodium content (since fermentation and storage took place in a salted brine). It should be emphasized that LAB can form biofilms which are rather complex polysaccharide-based matrices; these films promote their adherence not only to the olive drupe surface but also onto the inner surface of industrial equipment. Biofilms usually buildup as a mixture of microorganisms including LAB, yeasts, and other bacteria (even pathogenic ones); this may explain why successive spontaneous fermentation can be carried out in the same equipment, year after year, even in the absence of deliberate inoculation [22,50,113].

Industrial-scale processing of table olives with LAB starters has, in general, met with success, in terms of improvement of safety and sensory quality of the final product, along with a faster and more standardized fermentation process, further to a reduction in commodity losses and lower costs of production, when compared to conventional manufacturing protocols.

Almost no reports on LAB starters, readily available at the commercial scale and specific for table olive fermentation were unfortunately found; the major companies enrolled in this business—chiefly headquartered in European producing countries, viz. Spain, Greece, and Italy (as discussed before), seconded by countries that export table olives to Europe, viz. Morocco, Turkey, and Egypt, appear to use their proprietary starters under industrial secrecy. Concerning starter-producing companies, Caldwell sells a starter culture for fresh vegetables, constituted by *L. plantarum*, *L. mesenteroides*, and *Pedicoccus acidilactici*—which apparently performs well also with table olives; whereas SaccoSystem sells Lyoflora V-3, and Chr. Hansen sells Bactoferm^®^ Vege-Start60 and Bactoferm^®^ Vege-Start2.0—both claimed to perform well with table olives and consisting of a single strain of *L. plantarum*. 

## 6. Conclusions

The thorough search conducted on fermentation of table olives reveals that most scientific publications originate in countries of the Mediterranean basin—especially Italy, Greece, Portugal, and Spain. This relates to increased market demand for value-added products, such as nutraceutical or probiotic ingredients, which may also support a premium price for table olives derived from it being an appealing functional food.

Irrespective of production scale, the dynamics prevailing in fermentation is determined by parameters that constrain metabolism, namely, indigenous olive microbiota itself; this depends, in turn, on cultivar, degree of ripeness, region of origin, and manufacturer practices.

This review has listed the various LAB isolated from table olives with reports published throughout the past two decades; attempts were made to correlate them to country of origin, cultivar, type of processing, and drupe color. Among the nine genera of LAB identified in the literature, *L. plantarum* and *L. pentosus* are the species reported most often; however, *L. brevis*, *L. coryniformis*, *L. paraplantarum,* and *Lc. mesenteroides* also appeared with notable frequency. More recently, novel microorganisms (included in the LAB group) have been detected throughout fermentation by resorting to more sophisticated instrumental means and metagenomic analysis. This tendency unfolds the extreme need for studies to improve knowledge regarding the microbiota profile and its evolution during table olive fermentation. Further fundamental studies are urged to determine the role played by these species, including both quality and safety issues and to relate them with other microorganisms alike in similar or complementary ecosystems (e.g., via metabolomics).

Statistical analysis proved that olive color, treatment method, and cultivar are directly related to country and that LAB species also correlate with cultivar; this anticipates a consumer trend toward purchase of table olives processed according to local, traditional methods (including adventitious microbiota). Some of those autochthonous strains/species of LAB are obvious candidates to formal starter cultures, because of the several advantages they may convey to the fermentation process. This includes unique sensory profiles, and similarity of larger-scale manufacture to traditional practices; and will likely contribute to an eventual PDO status which by itself guarantees a premium price on the market. 

Finally, starter culture performance should as an advantage be tested directly at the industrial scale, rather than on the bench-scale followed by extrapolation to the larger scale. On the other hand, starter cultures specific for table olive production are far from being easily accessible on the market—which raises a hardship in their manufacture when compared to other fermented food products.

## Figures and Tables

**Table 1:** Trade preparations of table olives. Dark grey indicates a required processing step, light grey indicates an optional step, and white indicates absence of such a step.

Method Name	Debittering Treatment			Fermentation		Brine Treatment	Drying			Oxidation Treatment		Final Polishing			
	Adventitious Fermentation	Alkaline, %NaOH	Water Washing	Spontaneous	Starter Culture	% Salt	Salt	Heat	None	Aeration + Iron Salts	None	Sterilization	Tight Packaging	Gross Open Sell	Acidified Water
Natural ^1^						8–10									
Treated ^2^		2–5				10–11									
Darkened by oxidation ^3^		1–2				8–10									
Dehydrated/ Shriveled															

^1^ When applied to black olives, may also be called Greek-style. ^2^ When applied to green olives: if fermentation occurs, may also be called Spanish-style (5–10% NaCl; if fermentation does not occur, may also be called Picholine-style. ^3^ Also known as Californian-style.

**Table 2:** *Lactobacillus* species isolated from different processing methods of table olives.

Species	Country	Processing Method	Olive Cultivar	Study Objective	Reference
*Lactobacillus* sp.	Spain	Natural green olives	Aloreña	Identification of microbial diversity	[46]
*Lactobacillus acidipiscis*	Turkey	- (black olives)	-	Halophilic LAB identification	[47]
		- (green olives)	-	Halophilic LAB identification	[47]
	Spain	Treated green olives (Spanish-style)	Manzanilla	Identification of microbial diversity and biogeography	[48]
*Lactobacillus alimentarius*	Turkey	- (black olives)	-	Halophilic LAB identification	[47]
*Lactobacillus brevis*	Algeria	Natural black olives	Chemlal	LAB identification; Technological characterization	[49]
		Natural black olives	Hamra	LAB identification; Technological characterization	[49]
		Natural black olives	Sigoise	LAB identification; Technological characterization	[49]
	Italy	Natural green olives	Bella di Cerignola	Identification of microbial diversity; Probiotic starter culture application	[50]
		Natural green olives	-	LAB identification	[51]
	Morocco	Treated green olives (Picholine-style)	Picholine	Selection of oleuropein-degrading LAB strains	[52]
	Turkey	Natural green olives	-	LAB identification; Identification of microbial diversity	[53]
		Natural black olives	Gemlik	Starter culture application	[54]
*Lactobacillus casei*	Algeria	Natural green olives	Sigoise	LAB identification	[55]
	Italy	Natural green olives	Bella di Cerignola	Identification of microbial diversity; Probiotic starter culture application	[50]
		- (green olives)	-	Identification of microbial diversity; Safety evaluation	[56]
		Natural black olives	Cellina di Nardò	LAB identification; Physico-chemical characterization	[57]
		Natural black olives	Itrana nera	LAB identification; Physico-chemical characterization	[57]
		Natural green olives	Nocellara del Belice	LAB identification; Physico-chemical characterization	[57]
		Natural green olives	Itrana bianca	LAB identification; Physico-chemical characterization	[57]
		Natural green olives	Giarraffa	(Probiotic) Starter culture application	[58]
		Natural green olives	Grossa di Spagna	(Probiotic) Starter culture application	[58]
		Natural green olives	Nocellara Etnea	Starter culture application	[32,59]
		Natural green olives	-	LAB identification	[51]
*Lactobacillus casei* ssp. *tolerens*	Algeria	Natural black olives	Chemlal	LAB identification; Technological characterization	[49]
		Natural black olives	Hamra	LAB identification; Technological characterization	[49]
		Natural black olives	Sigoise	LAB identification; Technological characterization	[49]
*Lactobacillus collinoides*	Italy	Natural green olives	Tonda di Cagliari	Identification of microbial diversity; Sensory analysis	[60]
*Lactobacillus coryniformis*	Greece	Natural black olives	Conservolea	Identification of microbial diversity; Evaluation of storage conditions	[61]
	Italy	Natural green olives	Bella di Cerignola	Identification of microbial diversity; Probiotic starter culture application	[50]
		Natural green olives	Geracese	Identification of microbial diversity; Starter culture application	[62]
		Treated green olives (Spanish-style)	Nocellara del Belice	Starter culture application	[63]
		Natural green olives	Nocellara del Belice	Improvement of table olives’ growing conditions and processing method	[27,64]
		Treated green olives (Spanish-style)	Nocellara del Belice	LAB identification; Probiotic characterization	[65]
		Treated green olives	Nocellara	Identification of microbial diversity; Sensory analysis	[66]
	Spain	Treated green olives (Spanish-style)	Manzanilla	Identification of microbial diversity	[33]
*Lactobacillus coryniformis* ssp. *coryniformis/torquens*	Spain	Treated green olives (Spanish-style)	Manzanilla	Identification of microbial diversity and biogeography	[48]
*Lactobacillus curvatus*	Algeria	Natural black olives	Chemlal	LAB identification; Technological characterization LAB identification; Technological characterization LAB identification; Technological characterization	[49]
		Natural black olives	Hamra		[49]
		Natural black olives	Sigoise		[49]
*Lactobacillus farciminis*	Turkey	- (green olives)	-	Halophilic LAB identification	[47]
*Lactobacillus fermentum*	Italy	Natural black olives	Peranzana	LAB identification; Physico-chemical characterization	[57]
		Natural green olives	Bella di Cerignola	LAB identification; Physico-chemical characterization	[57]
*Lactobacillus helveticus*	Italy	Natural black olives	Cellina di Nardò	LAB identification; Physico-chemical characterization	[57]
*Lactobacillus mali*	Italy	Natural green olives	Bella di Cerignola	Identification of microbial diversity; Probiotic starter culture application	[50]
*Lactobacillus namurensis*	Turkey	- (black olives)	-	Halophilic LAB identification	[47]
*Lactobacillus oligofermentans*	Italy	Treated green olives (Spanish-style)	Nocellara del Belice	LAB identification; Probiotic characterization	[65]
*Lactobacillus paracasei*	Algeria	Natural green olives	Sigoise	LAB identification	[55]
	Italy	Natural green olives	Bella di Cerignola	Identification of microbial diversity; Probiotic starter culture application	[50]
		Natural green olives	Giarraffa	(Probiotic) Starter culture application	[58]
		Natural green olives	Nocellara Etnea	Starter culture application	[59]
	Spain	Treated green olives (Spanish-style)	Manzanilla	Identification of microbial diversity	[33,67]
*Lactobacillus paracasei* ssp. *paracasei*	Greece	Treated green olives (Spanish-style)	Conservolea	LAB identification	[68]
		Treated green olives (Spanish-style)	Halkidiki	LAB identification	[68]
*Lactobacillus paracollinoides*	Italy	Natural green olives	Geracese	Identification of microbial diversity; Starter culture application	[62]
	Spain	Treated green olives (Spanish-style)	-	Identification of ascorbic acid and potassium sorbate degrading LAB	[69]
		Natural green olives	Aloreña	Identification of microbial diversity	[46]
*Lactobacillus paracollinoides/collinoides*	Spain	Treated green olives (Spanish-style)	Manzanilla	Identification of microbial diversity and biogeography	[33,48,67]
*Lactobacillus parafarraginis*	Spain	Treated green olives (Spanish-style)	-	Identification of ascorbic acid and potassium sorbate degrading LAB	[69]
		Treated green olives (Spanish-style)	Manzanilla	Identification of microbial diversity and biogeography	[33,48,67]
		Treated green olives (Spanish-style)	Manzanilla	Identification of microbial diversity	[70]
		Treated green olives (Spanish-style)	Gordal	Identification of microbial diversity	[70]
*Lactobacillus paraplantarum*	Greece	Natural black olives	Conservolea	LAB identification	[68]
		Natural black olives	Kalamata	LAB identification	[68]
		Natural black olives	Conservolea	Identification of microbial diversity; Evaluation of storage conditions	[61]
	Italy	- (green olives)	-	Identification of microbial diversity; Safety evaluation	[56]
		Natural green olives	Tonda di Cagliari	Identification of microbial diversity; Sensory analysis	[60]
	Portugal	Natural black olives	Galega	Probiotic characterization	[71]
	Spain	Natural green olives	Arbequina	Identification of microbial diversity	[72]
		Treated green olives (Spanish-style)	Manzanilla	LAB identification; Probiotic characterization	[73]
		Treated green olives (Spanish-style)	Manzanilla	Identification of microbial diversity; Identification of LAB species	[33,67]
*Lactobacillus pentosus*	Greece	Natural black olives	Conservolea	LAB identification	[68]
		Natural black olives	Kalamata	LAB identification	[68]
		Treated green olives (Spanish-style)	Halkidiki	Starter culture application	[74]
		Treated green olives (Spanish-style)	Conservolea	LAB identification LAB identification	[68]
		Treated green olives (Spanish-style)	Halkidiki		[68]
		Treated green olives (Spanish-style)	Halkidiki	Shelf life evaluation	[75]
		Treated green olives (Spanish-style)	Halkidiki	Identification of microbial diversity (biofilm on fermentation vessels)	[76]
		Natural black olives	Conservolea	Identification of microbial diversity; Evaluation of storage conditions	[61]
		Natural black olives	Conservolea	Identification of microbial diversity; Starter culture application	[77]
	Italy	Natural green olives	Bella di Cerignola	Identification of microbial diversity; Probiotic starter culture application	[50]
		Natural black olives	Cellina di Nardò	LAB identification; Physico-chemical characterization	[57]
		Natural black olives	Itrana nera	LAB identification; Physico-chemical characterization	[57]
		Natural black olives	Peranzana	LAB identification; Physico-chemical characterization	[57]
		Natural green olives	Nocellara del Belice	LAB identification; Physico-chemical characterization	[57]
		Natural green olives	Itrana bianca	LAB identification; Physico-chemical characterization	[57]
		Natural green olives	Bella di Cerignola	LAB identification; Physico-chemical characterization	[57]
		Natural green olives	Bella di Cerignola	Identification of microbial diversity	[78]
		- (green olives)	-	Identification of microbial diversity; Safety evaluation	[56]
		Natural green olives	Tonda di Cagliari	Identification of microbial diversity; Sensory analysis	[60]
		Treated green olives (Spanish-style)	Nocellara del Belice	Starter culture application	[63]
		Treated green olives (Spanish-style)	Bella di Cerignola	Identification of microbial diversity	[78]
		Natural green olives	Giarraffa	(Probiotic) Starter culture application	[58]
		Natural green olives	Grossa di Spagna	(Probiotic) Starter culture application	[58]
		Natural green olives	Nocellara del Belice	Improvement of table olives’ growing conditions; Mechanical harvest evaluation	[64,79]
		Treated green olives (Spanish-style)	Nocellara del Belice	LAB identification; Probiotic characterization	[65]
		Natural green olives	Nocellara Etnea	Starter culture application; Safety evaluation; Sensory analysis	[80]
		Natural green olives	Nocellara Etnea	Starter culture application	[32,59]
		Treated green olives	Nocellara Etnea	Identification of microbial diversity; Evaluation of NaOH treatment	[81]
		Treated green olives	Nocellara	Identification of microbial diversity; Sensory analysis	[66]
	Morocco	Treated green olives(Picholine-style)	Picholine	Selection of oleuropein-degrading LAB strains	[52]
	Portugal	Natural black olives (in brine)	Galega	Identification of microbial diversity; Chemical characterization; Sensory analysis	[82]
	Spain	Natural green olives	Arbequina	Identification of microbial diversity	[24,72]
		Natural green olives	Aloreña	LAB identification; Technological and probiotic characterization; Influence of salt concentrations on shelf life	[73,83,84]
		Natural green olives	Gordal	Identification of microbial diversity on biofilms	[85]
		Natural green olives	Gordal	LAB identification; Probiotic characterization	[73]
		Natural green olives	Manzanilla	LAB identification; Probiotic characterization	[73]
		Treated green olives (Spanish-style)	Gordal	LAB identification; Probiotic characterization	[73]
		Treated green olives (Spanish-style)	-	Identification of ascorbic acid and potassium sorbate degrading LAB	[69]
		Treated green olives (Spanish-style)	Manzanilla	LAB identification; Probiotic characterization; Influence of salt concentrations during fermentation	[73,86]
		Treated green olives (Spanish-style)	Manzanilla	Identification of microbial diversity and LAB species and biogeography	[33,48,67]
		Natural black olives (in brine)	Manzanilla	Influence of salt concentrations in packaging brine	[87]
		Treated green olives (Spanish-style)	Manzanilla	LAB identification; Selection of a multifunctional starter culture	[88]
		Treated green olives (Spanish-style)	Gordal	LAB identification; Selection of a multifunctional starter culture	[88]
		Treated green olives (Spanish-style)	Aloreña	LAB identification; Selection of a multifunctional starter culture	[88]
		Natural green olives	Manzanilla	LAB identification; Selection of a multifunctional starter culture	[88]
		Natural green olives	Gordal	LAB identification; Selection of a multifunctional starter culture	[88]
		Natural green olives	Aloreña	LAB identification; Selection of a multifunctional starter culture	[88]
*Lactobacillus pentosus/plantarum*	Spain	Natural green olives	Aloreña	Identification of microbial diversity	[46]
*Lactobacillus plantarum*	Algeria	Natural black olives	Chemlal	LAB identification; Technological characterization LAB identification; Technological characterization LAB identification; Technological characterization	[49]
		Natural black olives	Hamra		[49]
		Natural black olives	Sigoise		[49]
		Natural green olives	Sigoise	Identification of microbial diversity	[89]
		Natural green olives	Sigoise	LAB identification	[55]
	Croatia	Treated green olives (Californian-style)	Oblica	Technological, physico-chemical characterization; LAB identification	[90]
	Greece	Natural black olives	Conservolea	LAB identification; Physico-chemical, probiotic characterization	[18,68,91]
		Natural black olives	Kalamata	LAB identification; Physico-chemical, probiotic characterization	[18,68,91]
		Treated green olives (Spanish-style)	Conservolea	LAB identification; Probiotic characterization	[68,91]
		Treated green olives (Spanish-style)	Halkidiki	LAB identification; Probiotic characterization	[68,91]
		Natural black olives	Conservolea	Development of new processing method	[92]
		Natural black olives	Conservolea	Identification of microbial diversity; Evaluation of storage conditions	[61]
	Italy	Natural green olives	Bella di Cerignola	Identification of microbial diversity; Probiotic starter culture application	[50]
		Treated green olives (Spanish-style)	Bella di Cerignola	Selection of a multifunctional starter culture	[93]
		- (green olives)	-	Identification of microbial diversity; Safety evaluation	[56]
		Natural black olives	Cellina di Nardò	Identification of microbial diversity and LAB; Physico-chemical characterization	[57]
		Natural black olives	Cellina di Nardò	Identification of microbial diversity; Physico-chemical characterization	[94]
		Natural black olives	Leccino	Identification of microbial diversity; Physico-chemical characterization	[94]
		Natural black olives	Itrana nera	LAB identification; Physico-chemical characterization	[57]
		Natural black olives	Peranzana	LAB identification; Physico-chemical characterization	[57]
		Natural green olives	Nocellara del Belice	LAB identification; Physico-chemical characterization	[57]
		Natural green olives	Itrana bianca	LAB identification; Physico-chemical characterization	[57]
		Natural green olives	Bella di Cerignola	LAB identification; Physico-chemical characterization	[57]
		Natural green olives	Bella di Cerignola	Identification of microbial diversity	[78]
		Natural green olives	Nocellara Etnea	Identification of microbial diversity; Starter culture application	[62]
		Natural green olives	Geracese	Identification of microbial diversity; Starter culture application	[62]
		Natural green olives	Tonda di Cagliari	Identification of microbial diversity; Sensory analysis	[60]
		Natural green olives	Giarraffa	(Probiotic) Starter culture application	[58]
		Natural green olives	Grossa di Spagna	(Probiotic) Starter culture application	[58]
		Treated green olives (Spanish-style)	Bella di Cerignola	Identification of microbial diversity	[78]
		Natural black olives	Cellina di Nardò	Development of new processing method	[92]
		Natural black olives	Leccino	Development of new processing method	[92]
		Natural green olives	Nocellara del Belice	Improvement of table olives’ growing conditions and processing method; Mechanical harvest evaluation	[27,64,79]
		Natural green olives	Nocellara Etnea	Starter culture application; Safety evaluation; Sensory analysis	[80]
		Natural green olives	Nocellara Etnea	Starter culture application	[32,59]
		Treated green olives	Nocellara Etnea	Identification of microbial diversity; Evaluation of NaOH treatment	[81]
		Natural green olives	-	LAB identification	[51]
	Morocco	Treated green olives (Spanish-style)	Picholine	Processing method improvement; Reducing bloater spoilage incidence	[95]
		Treated green olives (Picholine-style)	Picholine	Selection of oleuropein-degrading LAB strains	[52]
	Portugal	Natural black olives (in brine)	Galega	Identification of microbial diversity; Chemical characterization; Sensory analysis	[82]
		Natural black olives	Galega	Probiotic characterization	[71]
	Spain	Natural green olives	Arbequina	Identification of microbial diversity; Influence of salt concentrations during fermentation; Influence of fruit ripeness	[23,24,72]
		Natural green olives	Aloreña	Influence of salt concentrations on shelf life	[83]
		Treated green olives (Spanish-style)	Manzanilla	LAB identification; Probiotic characterization	[73]
		Treated green olives (Spanish-style)	Manzanilla	Identification of microbial diversity and LAB species	[33,48,67]
		Natural black olives (in brine)	Manzanilla	Influence of salt concentrations in packaging brine	[87]
		Treated green olives (Spanish-style)	Manzanilla	LAB identification; Selection of a multifunctional starter culture	[88]
		Treated green olives (Spanish-style)	Gordal	LAB identification; Selection of a multifunctional starter culture	[88]
		Natural green olives	Manzanilla	LAB identification; Selection of a multifunctional starter culture	[88]
		Natural green olives	Gordal	LAB identification; Selection of a multifunctional starter culture	[88]
		Natural green olives	Aloreña	LAB identification; Selection of a multifunctional starter culture	[88]
	Tunisia	-	-	Identification of microbial diversity	[96]
	Turkey	- (black olives)	-	Halophilic LAB identification	[47]
		- (green olives)	-	Halophilic LAB identification	[47]
		Natural green olives	-	LAB identification; Identification of microbial diversity	[53]
*Lactobacillus rapi*	Spain	Treated green olives (Spanish-style)	-	Identification of ascorbic acid and potassium sorbate degrading LAB	[69]
		Treated green olives (Spanish-style)	Manzanilla	Identification of microbial diversity and biogeography	[33,48,67]
*Lactobacillus rhamnosus*	Algeria	Natural green olives	Sigoise	LAB identification	[55]
	Italy	Natural green olives	Nocellara Etnea	Starter culture application; Safety evaluation; Sensory analysis	[80]
	Spain	Treated green olives (Spanish-style)	Manzanilla	Identification of microbial diversity and biogeography	[33,48,67]
*Lactobacillus sanfranciscensis*	Spain	Treated green olives (Spanish-style)	Manzanilla	Identification of microbial diversity	[70]
*Lactobacillus vaccinostercus*	Italy	Natural green olives	Bella di Cerignola	Identification of microbial diversity; Probiotic starter culture application	[50]
*Lactobacillus vaccinostercus/suebicus*	Spain	Natural green olives	Aloreña	Identification of microbial diversity	[46]
*Lactobacillus veridesens*	Algeria	Natural black olives	Chemlal	LAB identification; Technological characterization LAB identification; Technological characterization LAB identification; Technological characterization	[49]
		Natural black olives	Hamra		[49]
		Natural black olives	Sigoise		[49]

**Table 3:** Lactic acid bacteria (LAB) species (except *Lactobacillus*) isolated from different processing methods of table olives.

Species	Country	Processing Method	Olive Cultivar	Study Objective	Ref.
*Aerococcus viridans/urinaeequi*	Spain	Treated green olives (Spanish-style)	Manzanilla	Identification of microbial diversity	[33,67,97]
*Enterococcus durans*	Algeria	Natural green olives	Sigoise	LAB identification	[55]
	Italy	Natural black olives	Cellina di Nardò	LAB identification; Physico-chemical characterization	[4]
		Natural green olives	Itrana bianca	LAB identification; Physico-chemical characterization	[4]
		Natural green olives	Bella di Cerignola	LAB identification; Physico-chemical characterization	[4]
*Enterococcus casseliflavus*	Italy	Treated green olives (Spanish-style)	Nocellara del Belice	LAB identification; Probiotic characterization	[65]
	Spain	Treated green olives (Spanish-style)	Manzanilla	Identification of microbial diversity and biogeography	[33,48,67]
*Enterococcus casseliflavus* species group ^1^	Italy	Natural green olives	Bella di Cerignola	Identification of microbial diversity; Probiotic starter culture application	[50]
*Enterococcus faecalis*	Algeria	Natural green olives	-	Identification of microbial diversity	[89]
		Natural green olives	Sigoise	LAB identification	[55]
	Italy	Treated green olives (Spanish-style)	Bella di Cerignola	Selection of a multifunctional starter culture	[93]
		- (green olives)	-	Identification of microbial diversity; Safety evaluation	[56]
	Spain	Treated green olives (Spanish-style)	Manzanilla	Identification of microbial diversity and biogeography	[48]
*Enterococcus faecium*	Algeria	Natural green olives	Sigoise	LAB identification	[55]
	Cyprus	Natural green olives	-	Technological and probiotic characterization	[98]
	Italy	Natural green olives	Tonda di Cagliari	Identification of microbial diversity; Sensory analysis	[60]
		Natural green olives	-	LAB identification	[51]
	Turkey	- (black olives)	-	Halophilic LAB identification	[47]
		- (green olives)	-	Halophilic LAB identification	[47]
*Enterococcus gallinarum*	Italy	Treated green olives (Spanish-style)	Nocellara del Belice	LAB identification; Probiotic characterization	[65]
*Enterococcus italicus*	Italy	Natural green olives	Bella di Cerignola	Identification of microbial diversity; Probiotic starter culture application	[50]
*Enterococcus olivae*	Spain	Treated green olives (Spanish-style)	Manzanilla	Identification of microbial diversity	[33,67,97]
*Lactococcus formosensis/garviae*	Spain	Treated green olives (Spanish-style)	Manzanilla	Identification of microbial diversity and biogeography	[48]
*Lactococcus lactis*	Algeria	Natural green olives	-	Identification of microbial diversity	[89]
		Natural green olives	Sigoise	LAB identification	[55]
*Lactococcus lactis/Lactobacillus coryniformis*	Spain	Natural green olives	Aloreña	Identification of microbial diversity	[46]
*Leuconostoc cremoris*	Turkey	Natural black olives	Gemlik	Starter culture application	[54]
*Leuconostoc mesenteroides*	Algeria	Natural black olives	Chemlal	LAB identification; Technological characterization	[49]
		Natural black olives	Hamra	LAB identification; Technological characterization	[49]
		Natural black olives	Sigoise	LAB identification; Technological characterization	[49]
	Greece	Natural black olives	Kalamata	Physico-chemical characterization	[18]
		Natural black olives	Conservolea	LAB identification	[68]
		Natural black olives	Kalamata	LAB identification	[68]
		Natural black olives	Kalamata	Development of new processing method	[92]
	Italy	Natural green olives	Bella di Cerignola	Identification of microbial diversity	[78]
		Treated green olives (Spanish-style)	Bella di Cerignola	Identification of microbial diversity	[78]
		Natural black olives	Leccino	LAB identification	[99]
		Natural green olives	Bella di Cerignola	Identification of microbial diversity; Probiotic starter culture application	[50]
		- (green olives)	-	Identification of microbial diversity; Safety evaluation	[56]
		Natural green olives	Grossa di Spagna	(Probiotic) Starter culture application	[58]
		Treated green olives (Spanish-style)	Nocellara del Belice	LAB identification; Probiotic characterization	[65]
	Portugal	Natural black olives (in brine)	Galega	Identification of microbial diversity; Chemical characterization; Sensory analysis	[82]
	Turkey	Natural green olives	-	LAB identification; Identification of microbial diversity	[53]
*Leuconostoc mesenteroides* ssp. *mesenteroides*	Greece	Natural black olives	Kalamata	Starter culture application	[100]
*Leuconostoc paramesenteroides*	Turkey	Natural black olives	Gemlik	Starter culture application	[54]
*Leuconostoc mesenteroides* ssp. *group* ^2^	Spain	Treated green olives (Spanish-style)	Manzanilla	Identification of microbial diversity and biogeography	[48]
*Leuconostoc pseudomesenteroides*	Greece	Natural black olives	Conservolea	LAB identification	[68]
		Natural black olives	Kalamata	LAB identification	[68]
	Italy	Natural black olives	Leccino	LAB identification	[99]
	Spain	Natural green olives	Aloreña	Technological characterization	[84]
*Pediococcus acidilactici*	Italy	- (green olives)	-	Identification of microbial diversity; Safety evaluation	[56]
	Spain	Treated green olives (Spanish-style)	Manzanilla	Identification of microbial diversity	[67]
*Pediococcus damnosus*	Turkey	Natural green olives	-	LAB identification; Identification of microbial diversity	[53]
*Pediococcus ethanolidurans*	Spain	Treated green olives (Spanish-style)	-	Identification of ascorbic acid and potassium sorbate degrading LAB	[69]
		Treated green olives (Spanish-style)	Manzanilla	Identification of microbial diversity and biogeography	[33,48,67]
	Greece	Natural black olives	Conservolea	Identification of microbial diversity; Evaluation of storage conditions	[61]
*Pediococcus parvulus*	Italy	- (green olives)	-	Identification of microbial diversity; Safety evaluation	[56]
		Natural green olives	Geracese	Identification of microbial diversity; Starter culture application	[62]
		Natural green olives	Tonda di Cagliari	Identification of microbial diversity; Sensory analysis	[60]
	Spain	Treated green olives (Spanish-style)	Manzanilla	Identification of microbial diversity and biogeography	[33,48,67]
		Natural green olives	Aloreña	Technological characterization	[84]
*Pediococcus pentosaceus*	Italy	Natural black olives	Leccino	LAB identification	[99]
		Natural green olives	Nocellara del Belice	Improvement of table olives’ growing conditions and processing method	[27,64]
	Morocco	Treated green olives (Picholine-style)	Picholine	Selection of oleuropein-degrading LAB strains	[52]
	Portugal	Natural black olives (in brine)	Galega	Microbial and chemical characterization	[82]
*Sporolactobacillus inulinus/terrae*	Spain	Treated green olives (Spanish-style)	Manzanilla	Identification of microbial diversity and biogeography	[33,48]
*Streptococcus thermophilus*	Italy	Natural green olives	Geracese	Identification of microbial diversity; Starter culture application	[62]
*Weissella paramesenteroides*	Italy	Natural green olives	Bella di Cerignola	Identification of microbial diversity; Probiotic starter culture application	[50]
	Spain	Treated green olives (Spanish-style)	Manzanilla	Identification of microbial diversity and biogeography	[48]
*Weissella paramesenteroides/hellenica*	Spain	Treated green olives (Spanish-style)	Manzanilla	Identification of microbial diversity	[33,67]

^1^ Not specified. ^2^ *Leuconostoc mesenteroides* ssp. *mesenteroides/dextranicum/cremoris/suionicum*.

**Table 4:** Cramer’s V correlations matrix for the LAB, olive color, method type, and country variables.

	*Lactobacillus* Species	Olive Color	Method Type	Country
*Lactobacillus* species	1.000	-		
Olive color	0.471	1.000	-	
Method type	0.433	0.949	1.000	-
Country	0.500	0.618	0.552	1.000

**Table 5:** Cramer’s V correlations matrix for the LAB, method type, and cultivar variables.

	*Lactobacillus* Species	Method Type	Cultivar
*Lactobacillus* species	1.000	-	
Method type	0.364	1.000	-
Cultivar	0.347	0.673	1.000
